# HO-1-modified umbilical cord MSCs alleviate pulmonary arterial hypertension by reducing inflammation and endothelial dysfunction

**DOI:** 10.1093/stcltm/szag036

**Published:** 2026-06-27

**Authors:** Riken Chen, Xing Chen, Limei Liang, Huan Li, Liping Xu, Dongjie Huang, Yong Liu, Deyi Zhou, Weilong Ye, Shuyue Zhou, Yihuan Su, Dekang Nie, Zhenzhen Zheng, Yan Deng

**Affiliations:** Department of Respiratory and Critical Care Medicine, Second Division, The Second Affiliated Hospital of Guangdong Medical University, 12 Minyou Road, Xiashan District, Zhanjiang, Guangdong 524003, P.R. China; Department of Ultrasound, The First Affiliated Hospital of Guangxi Medical University, Nanning, Guangxi, China; Clinical Experiment Center, Guangxi Medical University, 22 Shuangyong Road, Nanning, Guangxi 530021, P.R. China; Department of Ultrasound, The First Affiliated Hospital of Guangxi Medical University, Nanning, Guangxi, China; Clinical Experiment Center, Guangxi Medical University, 22 Shuangyong Road, Nanning, Guangxi 530021, P.R. China; Department of Respiratory and Critical Care Medicine, Second Division, The Second Affiliated Hospital of Guangdong Medical University, 12 Minyou Road, Xiashan District, Zhanjiang, Guangdong 524003, P.R. China; Department of Ultrasound, The First Affiliated Hospital of Guangxi Medical University, Nanning, Guangxi, China; Clinical Experiment Center, Guangxi Medical University, 22 Shuangyong Road, Nanning, Guangxi 530021, P.R. China; Department of Respiratory and Critical Care Medicine, Second Division, The Second Affiliated Hospital of Guangdong Medical University, 12 Minyou Road, Xiashan District, Zhanjiang, Guangdong 524003, P.R. China; Department of Respiratory and Critical Care Medicine, Second Division, The Second Affiliated Hospital of Guangdong Medical University, 12 Minyou Road, Xiashan District, Zhanjiang, Guangdong 524003, P.R. China; Mudanjiang Medical University, 169 Zhongyang Street, Mudanjiang, Heilongjiang 157011, P.R. China; Department of Respiratory and Critical Care Medicine, Second Division, The Second Affiliated Hospital of Guangdong Medical University, 12 Minyou Road, Xiashan District, Zhanjiang, Guangdong 524003, P.R. China; Department of Respiratory and Critical Care Medicine, Second Division, The Second Affiliated Hospital of Guangdong Medical University, 12 Minyou Road, Xiashan District, Zhanjiang, Guangdong 524003, P.R. China; Department of Respiratory and Critical Care Medicine, Second Division, The Second Affiliated Hospital of Guangdong Medical University, 12 Minyou Road, Xiashan District, Zhanjiang, Guangdong 524003, P.R. China; Department of Respiratory and Critical Care Medicine, Second Division, The Second Affiliated Hospital of Guangdong Medical University, 12 Minyou Road, Xiashan District, Zhanjiang, Guangdong 524003, P.R. China; Department of Respiratory and Critical Care Medicine, Second Division, The Second Affiliated Hospital of Guangdong Medical University, 12 Minyou Road, Xiashan District, Zhanjiang, Guangdong 524003, P.R. China; Department of Ultrasound, The First Affiliated Hospital of Guangxi Medical University, 6 Shuangyong Road, Nanning, Guangxi 530021, P.R. China; Department of Respiratory and Critical Care Medicine, Second Division, The Second Affiliated Hospital of Guangdong Medical University, 12 Minyou Road, Xiashan District, Zhanjiang, Guangdong 524003, P.R. China; Department of Ultrasound, The First Affiliated Hospital of Guangxi Medical University, Nanning, Guangxi, China

**Keywords:** pulmonary arterial hypertension, mesenchymal stem cells, HO-1, oxidative stress, MAPK pathway

## Abstract

**Objective:**

To evaluate the efficacy of MSCs-HO-1 in pulmonary arterial hypertension (PAH) and explore the underlying mechanisms.

**Methods:**

HO-1 expression and localization in lung tissues and vessels were assessed using spatial transcriptomics and single-cell RNA sequencing analyses of human PAH. MSCs-HO-1 were intravenously administered in rat and mouse PAH models (MCT-induced and SuHx). Hemodynamics (RVSP), right ventricular hypertrophy index (RVHI), survival rate, and vascular remodeling were assessed by HE staining and α-SMA immunostaining. Inflammatory cytokines (IL-1β, IL-6, TNF-α, IL-18, IL-10, TGF-β, IL-4, IL-1Ra), ROS levels, and endothelial molecules [nitric oxide (NO) and prostaglandin I2 (PGI_2_)] were measured. RNA sequencing (RNA-seq) of PAECs was followed by pathway analysis and MAPK validation.

**Results:**

HO-1 was downregulated in PAH patients and models, mainly in the vascular endothelium. MSCs-HO-1 significantly reduced RVSP, RVHI, vascular remodeling, and improved survival compared to unmodified MSCs and HO-1 alone. MSCs-HO-1 inhibited pro-inflammatory cytokines (IL-1β, IL-6, TNF-α, IL-18), increased anti-inflammatory factors (IL-10, TGF-β, IL-4, IL-1Ra), reduced ROS, and restored NO/PGI_2_. PAEC migration and proliferation abnormalities were corrected. RNA-seq revealed multiple synergistic pathways, with MAPK playing a key role in endothelial protection.

**Conclusion:**

MSCs-HO-1 enhances endothelial function and pulmonary vascular remodeling by modulating antioxidant and immune responses, restoring NO-PGI_2_ signaling, and suppressing MAPK-mediated inflammation, providing more stable and significant effects than MSCs or HO-1 alone.

## Graphical abstract

**Graphical Abstract:**
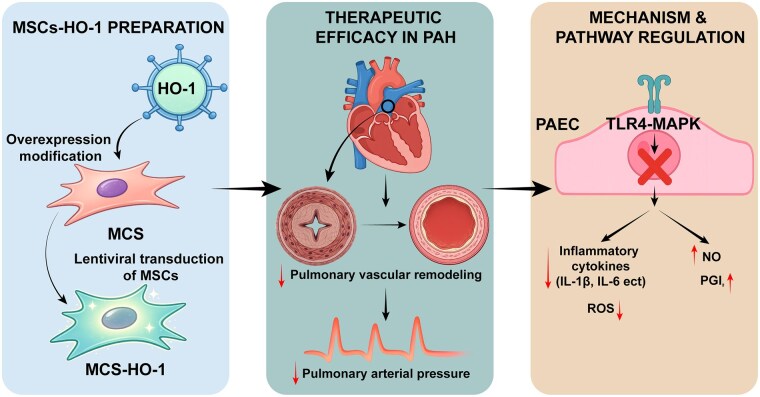

Significance statement Pulmonary arterial hypertension is a severe disease that damages the blood vessels of the lungs and can lead to heart failure. Current treatments mainly slow disease progression but rarely repair vascular injury. This study shows that genetically enhanced stem cells can reduce inflammation and oxidative stress, improve vascular function, and limit vascular remodeling in experimental models. These findings highlight the potential of modified stem cells as a promising strategy to repair pulmonary vascular damage and improve outcomes in this life-threatening disease.

## Introduction

Pulmonary arterial hypertension (PAH) is a severe pulmonary vascular disease characterized by PAEC dysfunction and abnormal proliferation of pulmonary artery smooth muscle cells (PASMCs) and pulmonary artery adventitial fibroblasts, which drives progressive occlusive pulmonary vascular remodeling, increased pulmonary arterial pressure, right ventricular failure, and ultimately death.^1–3^ Current PAH-targeted therapies primarily act on the endothelin pathway, the cyclic guanosine monophosphate pathway, and prostacyclin signaling; however, these agents merely relieve symptoms and delay disease progression, have limited capacity to reverse vascular remodeling, and show suboptimal efficacy in patients with advanced disease.^4–7^

In the pathogenesis of PAH, aberrant activation of inflammation and oxidative stress is recognized as a key driving force. Pro-inflammatory cytokines, including interleukin-18 (IL-18), interleukin-6 (IL-6), and interleukin-1 beta (IL-1β), promote vascular cell proliferation and migration, induce pulmonary artery endothelial cell (PAEC) apoptosis, and disrupt vascular homeostasis.^8–10^ Excessive reactive oxygen species (ROS) generation not only causes cellular injury but also amplifies inflammatory responses by activating inflammasomes such as NLRP3 and signaling pathways including nuclear factor kappa-light-chain-enhancer of activated B cells (NF-κB).^11–13^ The interaction between inflammation and oxidative stress forms a vicious cycle that accelerates vascular remodeling and elevates pulmonary arterial pressure. Therefore, targeting both inflammation and oxidative stress is considered a promising therapeutic strategy for PAH. Mesenchymal stem cells (MSCs) have gained increasing attention in regenerative medicine because of their immunomodulatory, paracrine, and tissue-repair properties. Among them, umbilical cord-derived MSCs (UC-MSCs) are particularly attractive due to their easy accessibility, low immunogenicity, and strong proliferative capacity.^14^ Recent studies have shown that UC-MSC transplantation provides cardioprotection and functional recovery through anti-inflammatory, antioxidant, and anti-apoptotic effects, as well as promotion of neovascularization, demonstrating therapeutic potential in dilated cardiomyopathy, myocardial infarction, and stroke.^15–17^ Although MSC-based therapy has shown promising potential in tissue repair, the early loss of transplanted cells in oxidative and inflammatory microenvironments substantially limits its therapeutic efficacy. In particular, mitochondrial oxidative stress-induced impairment of cell survival has emerged as a critical barrier to optimizing MSC-based strategies.^18^ In PAH, characterized by persistent inflammation, endothelial dysfunction, and oxidative stress, these limitations may be further exacerbated. However, whether UC-MSCs can modulate these processes and delay disease progression remains unclear.

Heme oxygenase-1 (HO-1), an antioxidant enzyme encoded by HMOX1, protects against oxidative stress and inflammation by scavenging ROS and inhibiting pyroptosis. HO-1 has been shown to reduce tissue injury and improve outcomes in inflammatory bowel disease, rheumatoid arthritis, and sepsis-induced acute lung injury.^19–21^

Based on this, we hypothesized that HO-1 modified UC-MSCs could serve as a potential therapy for PAH and exhibit greater efficacy than unmodified UC-MSCs. Their effects may be associated with reduced inflammation and oxidative stress in PAECs and subsequent improvement of endothelial dysfunction. This study aims to provide mechanistic evidence and support a potential cell-based therapeutic strategy targeting inflammation and endothelial dysfunction in early and intermediate stages of PAH.

## Methods

### Analysis of a public PAH dataset

This study utilized the GSE256539 dataset from the GEO database, which includes spatial transcriptomic data from lung tissues of 11 IPAH patients and 5 controls. Based on histopathological features, multiple regions of interest (ROIs) were selected for transcriptome sequencing, totaling 211 samples (149 IPAH ROIs and 62 control ROIs). Gene annotation was performed using the GPL30991 platform file, and data processing was done in R (version 4.3.2). Differentially expressed genes (DEGs) were identified with LogFC > 0 and *P *< .05 for upregulated genes, and LogFC < 0 and *P *< .05 for downregulated genes. DEGs were visualized using volcano plots.

### Collection of clinical samples from patients with IPAH

Lung and pulmonary vascular samples were collected from 30 patients with idiopathic pulmonary arterial hypertension diagnosed by echocardiography and right heart catheterization at the Second Affiliated Hospital of Guangdong Medical University. Thirty patients with pulmonary nodules but without pulmonary arterial hypertension served as controls. Inclusion criteria and clinical characteristics are shown in Supplementary Tables S1 and S2. All participants provided written informed consent, and the study was approved by the Ethics Committee (No. PJKT2025-126) in accordance with the Declaration of Helsinki.

### Establishment of PAH animal models

Male Sprague–Dawley rats (6-8 weeks, 200-250 g; Guangdong Medical University Animal Center) were randomly assigned to control or PAH groups (*n* = 5). Early PAH was induced by SU5416 plus hypoxia (SuHx; SU5416 20 mg/kg with 10% O_2_ for 3 weeks)^22^ or monocrotaline (MCT; 60 mg/kg for 3 weeks).^23^ Controls received saline under normoxia. A mouse PAH model was also established using C57BL/6 mice (6-8 weeks, 20-25 g) treated with MCT (50 mg/kg for 3 weeks),^24^ with saline-treated mice as controls. All procedures were approved by the Guangdong Medical University Ethics Committee (IACUC-202409815-105).

### Culture of human umbilical cord-derived mesenchymal stem cells

Human umbilical cord-derived mesenchymal stem cells (hUC-MSCs) were purchased from Cyagen Biosciences (HUXUC-01001) and cultured in DMEM/F12 (Gibco) supplemented with 10% FBS (Gibco) and 1% penicillin–streptomycin (Sigma-Aldrich) at 37 °C with 5% CO_2_. Cells were passaged or cryopreserved at 80% confluence.

### Preparation of HO-1 overexpressing virus

A three-plasmid system (PxpAx2, PMD2.G, and pLVX-Puro-GFP-HO-1) was used to generate lentiviral particles. PxpAx2 provided structural and helper proteins, PMD2.G encoded VSV-G envelope protein, and pLVX-Puro-GFP-HO-1 contained HO-1 and GFP. 293T cells were co-transfected with the plasmids at a 3:2:5 ratio using Lipofectamine™ 3000. After 48 h, the culture supernatant was collected, filtered, and concentrated by ultrafiltration to 1 mL.

### Lentiviral transduction of hUC-MSCs

hUC-MSCs at 70%-80% confluence were infected with HO-1–expressing lentivirus with a transduction enhancer. After 24 h, the medium was replaced with fresh DMEM. Puromycin (1 μg/mL) was added at 48 h to select stable clones, and HO-1 expression in MSCs-HO-1 was confirmed by real-time quantitative polymerase chain reaction (RT-qPCR).

### *In vivo* biodistribution and homing assay

To evaluate the *in vivo* retention of transplanted cells, MSCs-HO-1 were labeled with 1,1′-dioctadecyl-3,3,3′,3′-tetramethylindotricarbocyanine iodide (Dir; Solarbio) and intravenously injected into MCT-induced PAH rat and mouse models. Twenty-four hours after transplantation, animals were sacrificed, and major organs including the brain, heart, lung, liver, spleen, and kidney were collected. Fluorescence signals in each organ were detected using a small-animal *in vivo* imaging system to assess cell retention.

### Single-cell RNA sequencing data analysis

The single-cell RNA sequencing (scRNA-seq) dataset GSE228644 (three controls and three PAH samples) was obtained from GEO with annotation based on GPL20301. Data were processed using Seurat (v5.0) for quality control, normalization, and batch correction. Highly variable genes were identified, followed by PCA, UMAP visualization, and cell annotation using SingleR with manual refinement. Differential expression and pseudotime trajectory analyses were performed using Monocle3 (v1.3.1).

### Animal experiments

Three animal models were used. (i) SD rat MCT model: PAH was induced by a single MCT injection (60 mg/kg). MSCs or MSCs-HO-1 (4 × 10^6^ cells in 0.5-1 mL PBS) were injected via the tail vein on day 7. (ii) SD rat chronic hypoxia model: Rats were exposed to 10% O_2_ for 3 weeks, with MSCs or MSCs-HO-1 administered on day 7 as above. (iii) C57BL/6J mouse MCT model: PAH was induced with MCT (60 mg/kg), followed by MSCs or MSCs-HO-1 injection (10^6^ cells in 100-200 μL PBS) on day 7. Animals were observed until day 21. Health status and survival were monitored, with randomization and blinding applied.

### Right heart catheterization

Under isoflurane anesthesia (4% induction, 2% maintenance), animals were intubated and ventilated (I: E = 1:1, 90 breaths/min, tidal volume 1.0 mL). After median sternotomy, a 3.5 mm pressure catheter was inserted into the right ventricle to record right ventricular pressure. RVSP was used as an indirect measure of pulmonary artery pressure, and mPAP as an additional hemodynamic parameter.

### Calculation of right ventricular hypertrophy index

After hemodynamic measurements, hearts were excised and atrial tissue removed. The right ventricle (RV) was separated from the left ventricle plus septum (LV+S) and weighed. Right ventricular hypertrophy index (RVHI) was calculated as RV/(LV+S).

### Histopathological assessment of pulmonary arteries

Lung tissues were fixed in 4% paraformaldehyde, paraffin-embedded, and sectioned at 4 μm. HE staining was performed to assess structural changes in small pulmonary arteries. Pulmonary arterioles with an external diameter (ED) of 50-100 μm were selected, and ED and internal diameter (ID) were measured. Medial wall thickness (WT%) and wall area (WA%) were calculated to evaluate pulmonary vascular remodeling. The formulas were as follows:


WT%=[(ED-ID)/ED]×100%;

$$\mathrm{WA}\%=[({\mathrm{ED}}^{2}-{\mathrm{ID}}^{2})/{\mathrm{ED}}^{2}]\times 100\%.$$

Vascular muscularization was further assessed by immunohistochemical (IHC) staining for α-smooth muscle actin (α-SMA, CST).

### Isolation of primary PAECs

After sacrifice, trachea, lungs, and heart were removed and placed in sterile PBS. Pulmonary arteries were dissected, flushed with PBS, and cut into 2 mm fragments, which were placed in a T25 flask and incubated for 2 h to allow adhesion. DMEM with 20% FBS was added to cover the explants. Once PAECs migrated out and reached >80% confluence, the medium was replaced, and cells were passaged for further experiments.

### Cell experiments

Primary PAECs were isolated from Control and PAH model rats as described in Section 2.12. Cells were divided into five groups: CON (control PAECs), PAH (PAH model PAECs), PAH+MSCs (PAH PAECs co-cultured with hUC-MSCs), PAH+MSCs-HO-1 (PAH PAECs co-cultured with HO-1-overexpressing hUC-MSCs), and PAH+HO-1 (PAH PAECs transduced with HO-1 lentivirus). For pathway validation, cells were divided into Con, PAH, PAH+MSCs-HO-1, PAH+MSCs-HO-1+solvent, and PAH+MSCs-HO-1+Anisomycin groups.^25^ Western blotting was used to detect inflammatory cytokines (IL-1β, TNF-α, IL-6, IL-18) and MAPK pathway proteins (p38, p-p38, ERK, p-ERK, JNK, p-JNK). In the co-culture experiments, PAECs and MSCs were co-cultured at a 2:1 ratio for 24 h. Cells were then gently digested and collected, and a short-term differential adhesion method was applied to remove rapidly adhering MSCs, thereby enriching PAECs. The purified cells were subsequently identified by immunofluorescence (IF) staining for the endothelial marker CD31 to confirm purity before being used for subsequent assays of inflammation, oxidative stress, and cellular function.

### RT-qPCR

Total RNA was extracted using TRIzol reagent (Invitrogen), and RNA purity was assessed with a Nano spectrophotometer (Thermo Fisher) with an A260/A280 ratio ≥1.8. One μg RNA was reverse-transcribed into cDNA using a Takara kit. qPCR was performed with SYBR Premix Ex Taq II (Takara) on an ABI 7500 system, with each reaction containing 10 μL 2× SYBR Mix, 0.4 μL primers, 2 μL cDNA, and water to 20 μL. Cycling conditions were 95 °C for 30 s, then, 40 cycles of 95 °C for 5 s and 60 °C for 30 s. *GAPDH* was used as the internal reference, and relative mRNA expression was calculated using the 2^−ΔΔCt method. Primer sequences are listed in Supplementary Table S3.

### RNA sequencing

Total RNA was extracted from treated PAECs (MCT-induced PAH, *n* = 5; MCT+MSCs-HO-1, *n* = 5) using the TRIzol method. RNA purity and integrity were assessed, and qualified samples underwent library construction and high-throughput sequencing on the Illumina NovaSeq 6000 platform. After quality control, clean reads were aligned to the reference genome, and gene expression levels were quantified. DEGs were identified (*P *< .05), and GO/KEGG enrichment analyses were performed to identify key biological processes and pathways.

### Western blot

Cells or tissue specimens were lysed in RIPA buffer with protease and phosphatase inhibitors, incubated on ice for 30 min, and centrifuged at 12 000*g* for 20 min at 4 °C. Protein concentrations were measured using a BCA assay kit (Beyotime). Equal protein amounts (20-40 μg) were mixed with loading buffer, denatured, and separated by SDS-PAGE, then transferred to PVDF membranes (Millipore). Membranes were blocked and incubated overnight at 4 °C with primary antibodies (antibody information is provided in Supplementary Table S4), followed by washing with TBST. After incubation with HRP-conjugated secondary antibodies, bands were visualized using ECL substrate and imaged. Band intensities were quantified using ImageJ, and protein levels were normalized to β-actin.

### TUNEL assay for detection of apoptosis

Cell apoptosis was assessed using a TUNEL kit (Beyotime). Cells were seeded on glass coverslips, fixed with 4% paraformaldehyde, and permeabilized with 0.1% Triton X-100. TUNEL solution was added and incubated at 37 °C for 1 h. After washing with PBS, nuclei were counterstained with DAPI. TUNEL-positive apoptotic cells with green fluorescence were observed, and the apoptotic rate was quantified.

### Immunofluorescence

IF staining was performed to detect HO-1 localization in PAECs. Cells and tissue sections were incubated overnight at 4 °C with primary antibodies against CD31 (1:200, Zhengneng Biotechnology, 347526) and HO-1 (1:100, Zhengneng Biotechnology, R24541), followed by Alexa Fluor 488/594 secondary antibodies (1:200) for 1 h and DAPI staining. Images were acquired by confocal microscopy, and co-localization was analyzed using ImageJ.

### Cell Counting Kit-8 assay

Cell viability was assessed using a Cell Counting Kit-8 (CCK-8) kit (Beyotime). After treatment, 10 μL of CCK-8 solution was added to each well, and cells were incubated for 2 h at 37 °C. Absorbance at 450 nm (OD450) was measured using a microplate reader to calculate cell viability.

### Wound-healing assay

Cells were seeded in six-well plates and grown to 90% confluence. A linear scratch was made with a 200 μL pipette tip, and detached cells were removed with PBS. Cells were cultured in serum-free DMEM, and wound images were captured at 0 and 24 h. Migration distance was measured using ImageJ.

### Transwell migration assay

Cell migration was evaluated using Transwell chambers (8 μm pores). Cells (1 × 10^4^) in serum-free medium were seeded in the upper chamber, with DMEM containing 2% FBS in the lower chamber. After 24 h, migrated cells were fixed, crystal violet stained, and counted in five random fields.

### ELISA for inflammatory markers

Inflammation-related cytokines were measured using a sandwich ELISA kit (Elabscience). Supernatants were centrifuged at 3000*g* for 15 min, and tissue samples were homogenized and centrifuged at 12 000*g* for 15 min. Samples (100 μL) were incubated on a pre-coated plate, and absorbance at 450 nm was measured. Cytokine concentrations were calculated from the standard curve and adjusted for dilution.

### Oxidative stress assays

#### ROS detection

Cells were treated as described, and supernatants were collected after 48 h. Nitric oxide (NO) levels were measured using a nitrate reductase assay kit (MEIMIAN). Samples were incubated with reaction solution at 37 °C for 10 min, and absorbance at 540 nm was measured. NO concentrations were calculated from a standard curve.

#### Nitric oxide detection

Cells were treated as described, and culture supernatants were collected after 48 h. NO levels were measured using a nitrate reductase kit (MEIMIAN). The supernatant was mixed with the reaction solution, incubated at 37 °C for 10 min, centrifuged, and absorbance at 540 nm was measured. NO concentrations were calculated from the standard curve.

#### Prostaglandin I2 detection

Cells were treated as for NO detection, and supernatants were collected after 48 h. Prostaglandin I2 (PGI_2_) levels were measured using a PGI_2_ ELISA kit (MEIMIAN). Standards and samples were added to the plate, incubated, washed, and incubated with chromogenic reagents. After reaction termination, absorbance at 450 nm was measured, and PGI_2_ concentrations were calculated from the standard curve.

#### Statistical analysis

Data are presented as mean ± SD. Statistical analysis was performed using GraphPad Prism 10.5. An independent *t*-test was used for two-group comparisons, and one-way ANOVA with LSD post hoc test was used for multiple groups (normal distribution). The Kruskal–Wallis test with Dunn’s *post hoc* test was used for non-normally distributed data. *P *< .05 was considered significant. *P < .05, **P < .01, ***P < .0001, ns *P *> .05.

## Results

### Reduced expression of HO-1 in patients with IPAH

**Figure 1:**
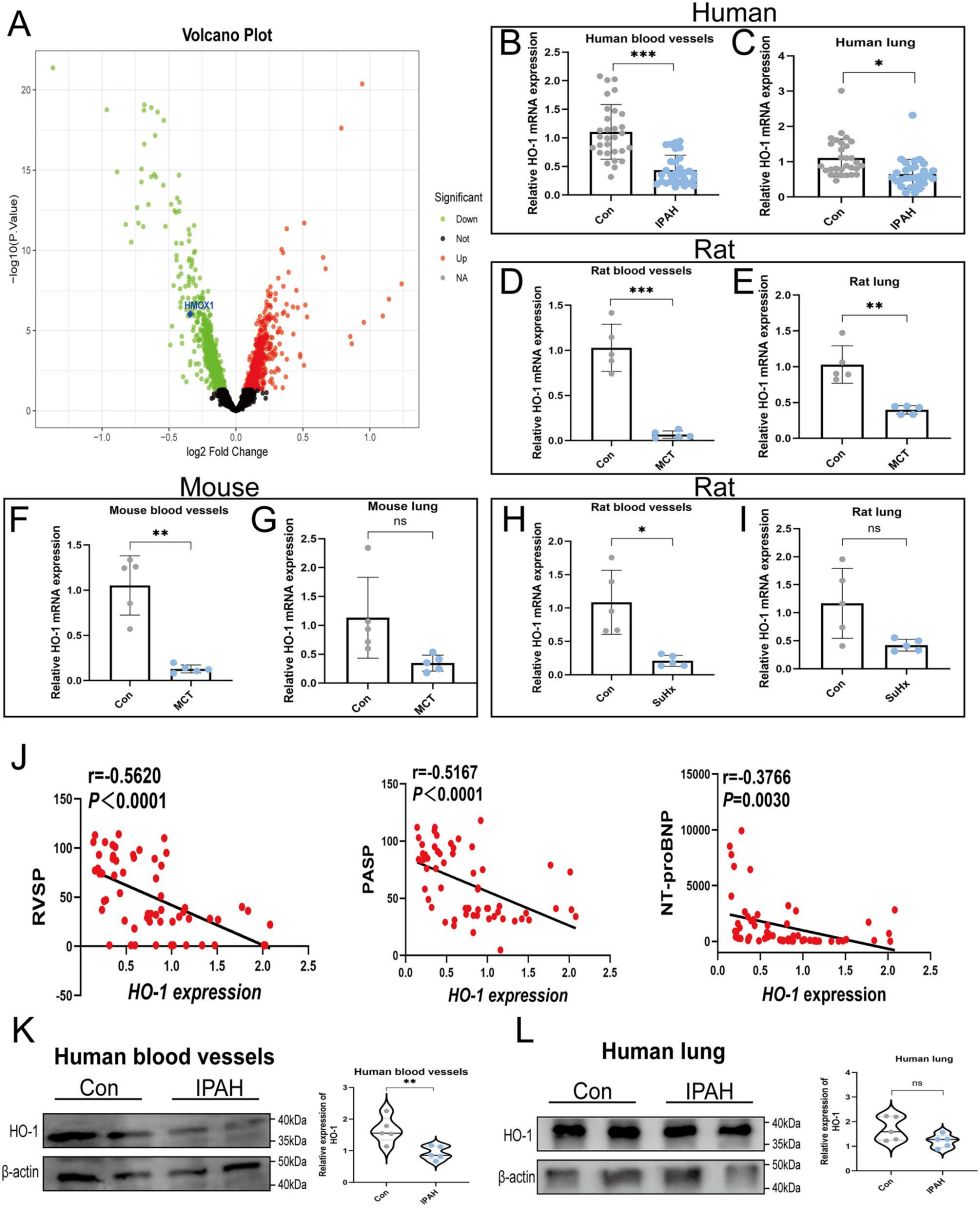
Decreased expression of HO-1 in patients with PAH and in experimental PAH models. (A) Volcano plot from spatial transcriptomic analysis of the GSE256539 dataset showing that *HO-1* (*HMOX1*) is significantly downregulated in PAH-related samples. (B, C) *HO-1* mRNA expression in human lung tissue and pulmonary vascular samples detected by RT-qPCR. (D, E) *HO-1* mRNA levels in vascular tissue (D) and lung tissue (E) from rats in the MCT-induced PAH model, as assessed by RT-qPCR. (F, G) *HO-1* mRNA levels in vascular tissue (F) and lung tissue (G) from mice in the MCT-induced PAH model, as assessed by RT-qPCR. (H, I) *HO-1* mRNA levels in vascular tissue (H) and lung tissue (I) from rats in the Su/Hx-induced PAH model, as assessed by RT-qPCR. (J) Correlation analysis between *HO-1* expression and clinical parameters in IPAH patients and control subjects. (K, L) HO-1 protein expression in human pulmonary vascular and lung tissue samples detected by Western blot. (K) Representative immunoblots and quantitative analysis of human pulmonary vascular samples. (L) Representative immunoblots and quantitative analysis of human lung tissue samples (Con: *n* = 5; IPAH: *n* = 5). Target proteins and loading controls were derived from the same membrane. Human samples: *n* = 20 per group; animal samples: *n* = 6 per group; all experiments were independently repeated three times. Differences were considered statistically significant at *P *< .05. *P < .05, **P < .01, ***P < .0001, ns *P *> .05.

Spatial transcriptomic analysis of the GSE256539 dataset (Figure 1A) showed significant downregulation of *HO-1* (*HMOX1*) in PAH samples. RT-qPCR in human PAH samples confirmed lower *HO-1* mRNA expression in both vascular (Figure 1B) and lung tissue (Figure 1C) compared to controls. In the rat MCT-induced PAH model, *HO-1* mRNA was reduced in both vascular (Figure 1D) and lung tissue (Figure 1E). In the mouse MCT model, HO-1 was significantly reduced in vascular tissue (Figure 1F), but not in lung tissue (Figure 1G). In the Su/Hx rat model, *HO-1* mRNA was lower in vascular tissue (Figure 1H), but not in lung tissue (Figure 1I). Correlation analysis showed that HO-1 expression was significantly negatively correlated with RVSP, PASP, and NT-proBNP levels in IPAH patients (Figure 1J). Reduced HO-1 expression was associated with increased disease severity. Western blot showed HO-1 protein downregulation in both pulmonary vascular and lung tissue from IPAH patients, though the reduction in lung tissue was not significant (Figure 1K and L). This contrasts with transcriptomic analysis, likely due to the small change in lung tissue, detectable only at the transcript level. These results suggest that HO-1 may play a key role in PAH-related pulmonary vascular injury.

### Anti-inflammatory phenotype of HO-1-overexpressing MSCs

**Figure 2:**
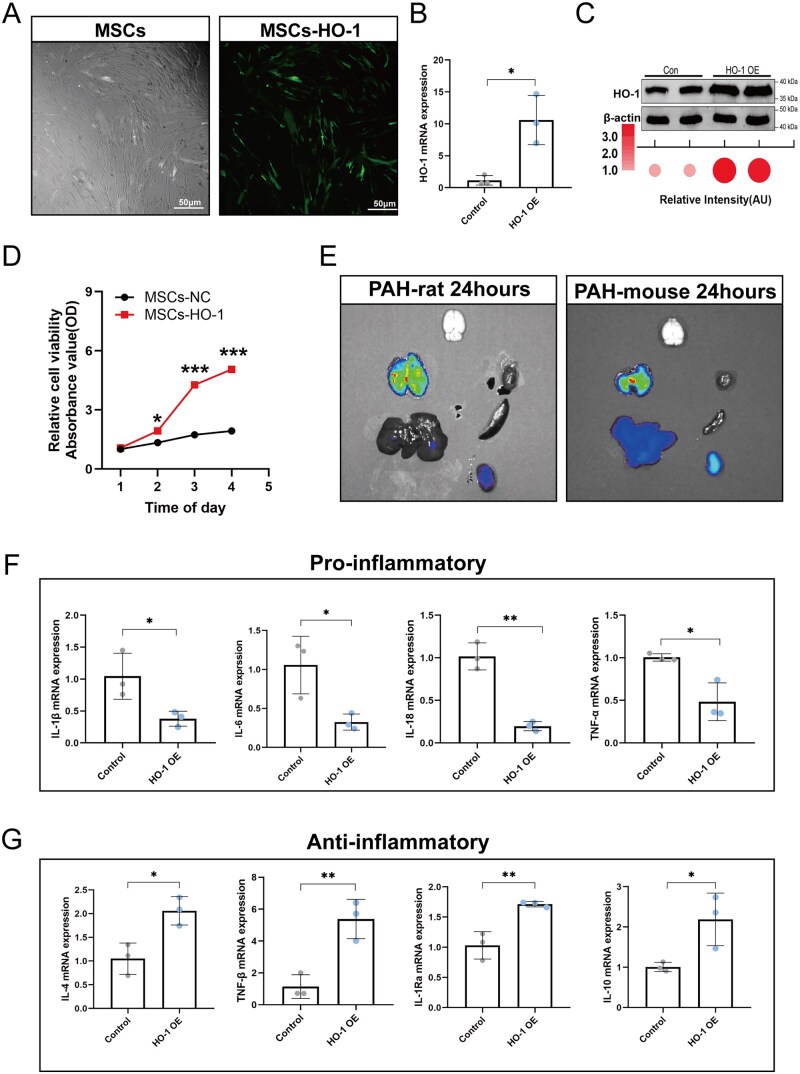
Generation of MSCs-HO-1 and validation of their anti-inflammatory properties. (A) Bright-field and IF images showing HO-1 expression in MSCs and MSCs-HO-1. Scale bar = 50 μm. (B) *HO-1* mRNA expression levels detected by RT-qPCR. (C) HO-1 protein expression detected by Western blot; target proteins and loading controls were obtained from the same membrane. (D) CCK-8 assay showing relative cell viability of MSCs-NC and MSCs-HO-1 over time. (E) *In vivo* fluorescence imaging showing pulmonary retention of transplanted cells in PAH rat and mouse models at 24 h post-injection. (F) RT-qPCR analysis of mRNA expression levels of pro-inflammatory cytokines (*IL-1β*, *IL-6*, *IL-18*, *TNF-α*). (G) RT-qPCR analysis of mRNA expression levels of anti-inflammatory cytokines (*IL-4*, *TNF-β*, *IL-1Ra*, *IL-10*). For cell experiments, *n* = 3 per group, and all experiments were independently repeated three times. Differences were considered statistically significant at *P *< .05. *P < .05, **P < .01, ***P < .0001, ns *P *> .05.

To assess whether HO-1 expression enhances the immunomodulatory capacity of MSCs, we established MSCs-HO-1. HO-1 overexpression was expected to boost MSC antioxidant capacity and reduce pro-inflammatory signaling. IF staining confirmed successful HO-1 transduction in MSCs-HO-1, with green fluorescence observed in MSCs-HO-1 but not in control MSCs (Figure 2A). RT-qPCR and Western blotting showed significant increases in *HO-1* mRNA and protein levels in MSCs-HO-1 (Figure 2B and C). CCK-8 assays demonstrated that HO-1 overexpression significantly enhanced MSC proliferation, suggesting improved *in vivo* survival potential (Figure 2D). *In vivo* fluorescence imaging further showed that MSCs-HO-1 were predominantly retained in the lungs at 24 h in PAH rat and mouse models, with the strongest signal observed in lung tissue (Figure 2E). These findings indicate that HO-1 modification promotes MSC survival and enrichment in diseased pulmonary tissue. HO-1 overexpression reduced pro-inflammatory cytokines (*IL-1β*, *IL-6*, *IL-18*, *TNF-α*) and increased anti-inflammatory cytokines (*IL-4*, *TNF-β*, *IL-1Ra*, *IL-10*; Figure 2F and G). These results suggest that HO-1 enhances MSCs’ anti-inflammatory and immunoregulatory functions.

### MSCs-HO-1 attenuate pulmonary hemodynamics, right ventricular dysfunction, and pulmonary vascular remodeling in multiple PAH models

**Figure 3:**
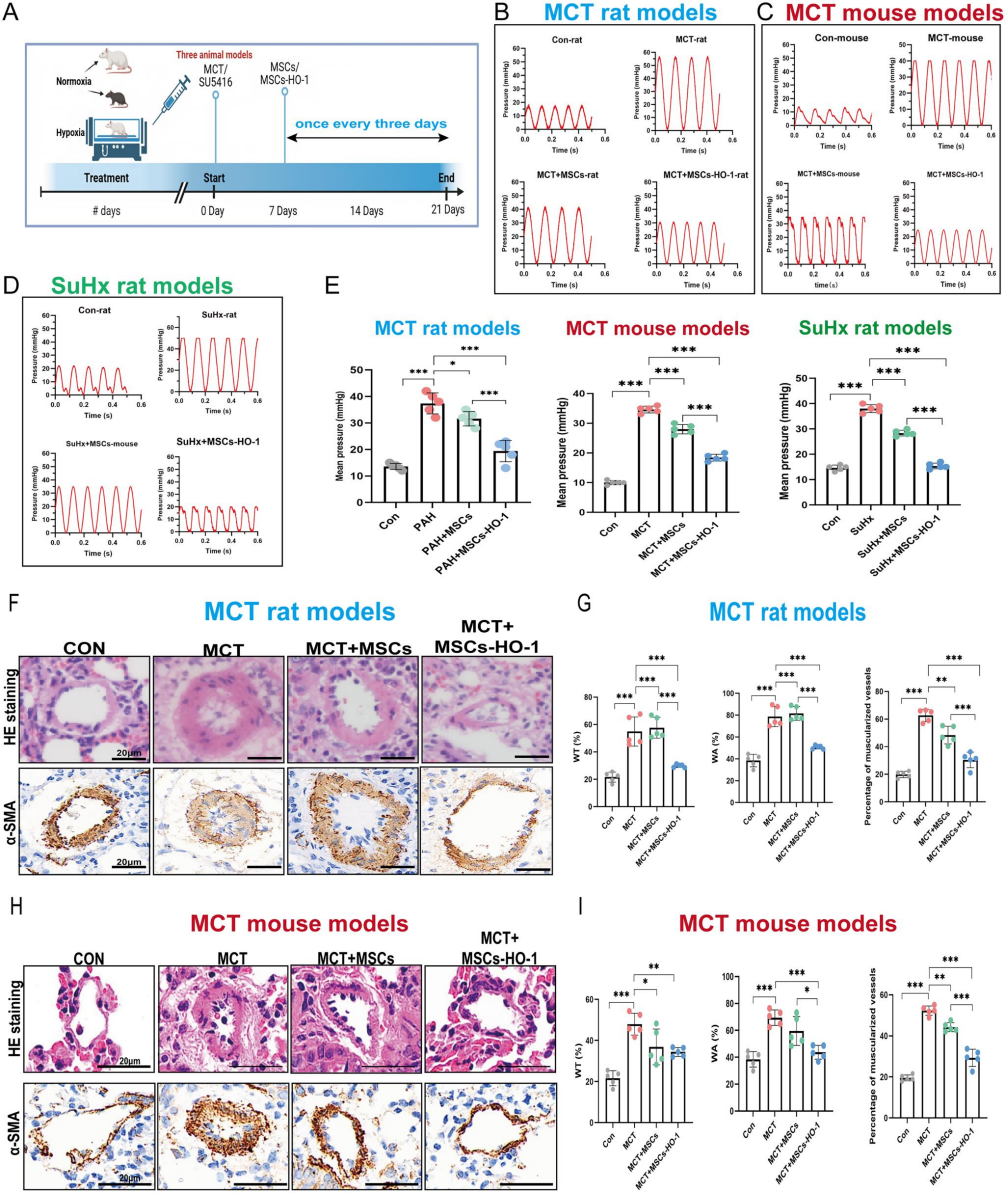
Hemodynamic and pulmonary vascular structural analysis in PAH animal models following MSCs-HO-1 intervention. (A) Schematic timeline of animal model development and intervention. (B-E) Hemodynamic parameters were assessed using right heart catheterization in MCT-induced PAH rat and mouse models. (B) Characteristic RVSP in the MCT rat model; (C) Characteristic RVSP in the MCT mouse model; (D) Characteristic RVSP in the hypoxic rat model; (E) Visualized mPAP images in the three animal models. (F-I) HE and α-SMA staining of lung tissue to observe changes in the thickness and muscularization of small pulmonary arteries. (F) Representative HE and α-SMA staining images of characteristic small pulmonary arteries in the MCT rat model; (G) Bar graph evaluating the wall thickness, WT, WA, and muscularization positive rate of small pulmonary arteries in the MCT rat model; (H) Representative HE and α-SMA staining images of characteristic small pulmonary arteries in the MCT mouse model; (I) Bar graph evaluating the wall thickness, WT, WA, and muscularization positive rate of small pulmonary arteries in the MCT mouse model. Animal experiments: *n* = 5 per group, with each experiment independently repeated three times. Differences were considered statistically significant at *P *< .05. *P < .05, **P < .01, ***P < .0001, ns *P *> .05.

**Figure 4:**
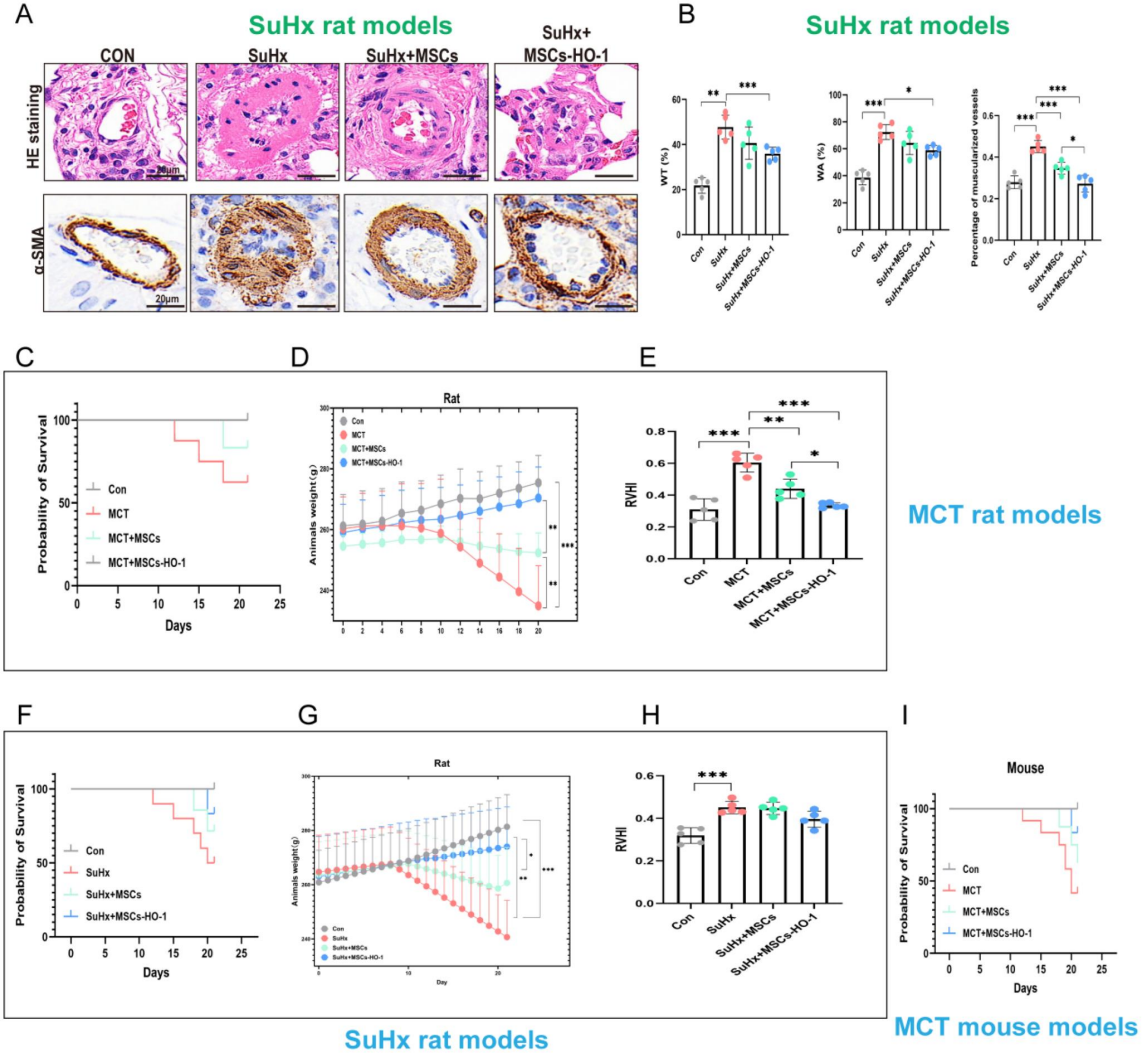
Validation and survival analysis of MSCs-HO-1 in different PAH models. (A, B) HE and α-SMA staining of lung tissue to observe changes in the thickness and muscularization of small pulmonary arteries. (A) Representative HE and α-SMA staining images of characteristic small pulmonary arteries in the SuHx mouse model; (B) Bar graph evaluating the wall thickness and muscularization positive rate of small pulmonary arteries in the SuHx mouse model. (C-E) Survival rate in each experimental group of the MCT rat model (C); body weight change curve (D); RVHI, representing right ventricular function assessment (E). (F-H) Survival rate in each experimental group of the SuHx rat model (F); body weight change curve (G); RVHI, representing right ventricular function assessment (H). (I) Survival rate in each experimental group of the MCT mouse model. Animal experiments: *n* = 5 per group, with each experiment independently repeated three times. Differences were considered statistically significant at *P *< .05. *P < .05, **P < .01, ***P < .0001, ns *P *> .05.

To assess the therapeutic effects of HO-1-overexpressing MSCs *in vivo*, we performed cell transplantation in MCT-induced and Su/Hx-induced PAH rat and mouse models (Figure 3A). MSCs-HO-1 treatment significantly reduced RVSP and mPAP compared to PAH model groups, indicating improved pulmonary arterial pressure (Figure 3B-E). The MSCs-HO-1 group showed more pronounced improvement than the unmodified MSCs group. Histological analysis revealed reduced medial thickening and vascular remodeling in the MSCs-HO-1 group (Figures 3F-I and 4A and B), with unmodified MSCs also improving, but to a lesser extent. RVHI further confirmed the improvement in right ventricular remodeling (Figure 4E and H). Survival analysis showed increased survival in the MSCs-HO-1 group, along with better clinical condition, including higher activity and less body weight loss (Figure 4C, D, F, G, and I). These results suggest that MSCs-HO-1 outperform unmodified MSCs in slowing PAH progression by improving pulmonary hemodynamics, inhibiting vascular remodeling, and reducing RV load.

### MSCs-HO-1 improves PAH by counteracting inflammation

After evaluating the therapeutic effects in three animal models (Figures 3 and 4), the MCT-induced PAH rat model showed the best outcome, so it was selected for further studies. Inflammation-related cytokine expression in lung tissue was assessed by RT-qPCR. Pro-inflammatory cytokines (*IL-1β*, *IL-6*, *IL-18*, *TNF-α*) were significantly elevated in the PAH group, indicating inflammation. MSC treatment reduced these cytokines, with a greater reduction in the MSCs-HO-1 group (Supplementary Figure S1A). Anti-inflammatory cytokines (*IL-4*, *TNF-β*, *IL-1Ra*, *IL-10*) were downregulated in PAH rats, but MSCs partially restored their levels. MSCs-HO-1 treatment further increased these cytokines compared to MSCs (Supplementary Figure S1B). These results confirm that HO-1 overexpression enhances MSCs’ anti-inflammatory effects, improving the inflammatory state in PAH.

### Downregulation of HO-1 expression is closely associated with PAEC dysfunction in PAH

**Figure 5:**
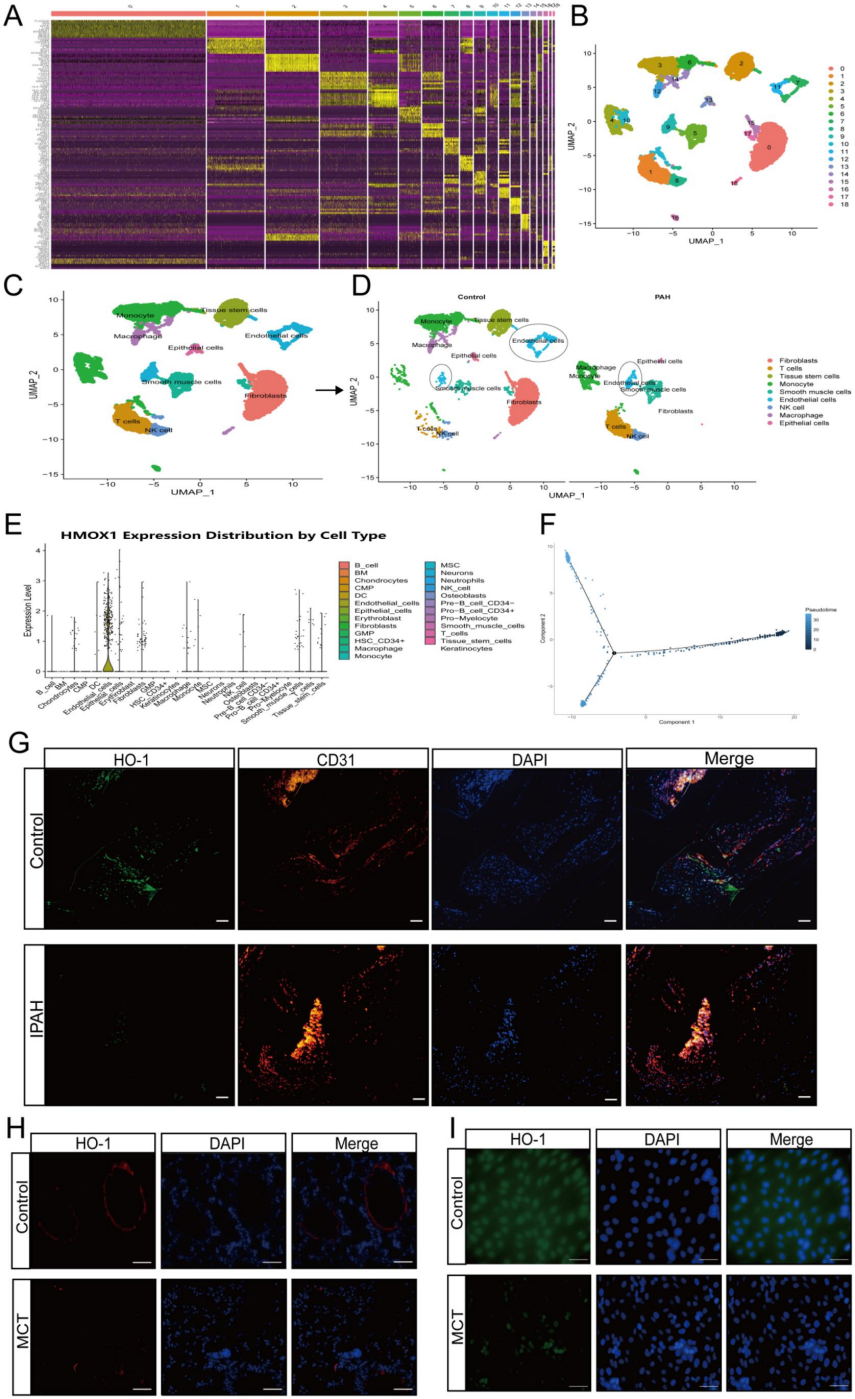
HO-1 expression characteristics in PAH based on scRNA-seq analysis and its IF localization detection. (A) Heatmap analysis of the publicly available scRNA-seq dataset GSE228644 from the GEO database (human pulmonary arterial tissue, including three normal controls and three PAH patients), comparing differential gene expression profiles across different cell clusters. (B) Single-cell clustering visualization, where each color represents a distinct cell cluster. (C, D) Annotation of major cell clusters based on marker genes, comparing the distribution of cell populations between the control and PAH groups. (E) Analysis of *HMOX1* (*HO-1* encoding gene) expression distribution across different cell types. (F) Pseudotime analysis evaluating the dynamic changes in the state of PAECs during the progression of PAH. (G) IF staining of lung vascular tissue sections from IPAH patients and non-PAH controls to detect HO-1 localization and expression, scale bar = 200 μm. (H) IF staining of lung tissue sections from MCT rats and controls to detect HO-1 localization and expression, scale bar = 20 μm. (I) IF staining of PAECs isolated from normal control and MCT-induced PAH rats to detect HO-1 expression, scale bar = 20 μm. IF localization experiments: *n* = 3 per group, with each experiment independently repeated three times. Differences were considered statistically significant at *P *< .05. *P < .05, **P < .01, ***P < .0001, ns *P *> .05.

To investigate the mechanisms of MSCs-HO-1 in PAH, we analyzed the GSE228644 scRNA-seq dataset from human pulmonary arterial tissue (3 controls, 3 PAH patients). The goal was to uncover the cellular mechanisms and pathways of MSCs-HO-1 in PAH treatment. Figure 5A shows a heatmap of differentially expressed genes, and Figure 5B displays UMAP clustering of cells, highlighting transcriptional differences. Figure 5C and D annotates major cell populations with significant differences between normal and PAH groups. The results indicate that in PAH, the overall number of PAECs is reduced, with an increase in PASMCs and inflammatory cell infiltration. This pathological change aligns with previous studies:^12^^,^^26^ during PAH progression, although some PAECs undergo apoptosis or detachment, the surviving PAECs fail to maintain normal homeostasis, resulting in significant functional imbalance. This imbalance in PAH is marked by abnormal activation, excessive proliferation, and features such as pro-inflammatory, pro-coagulant, and endothelial-mesenchymal transition. Figure 5E shows *HMOX1* expression, highest in PAECs. Figure 5F indicates that PAECs exhibit dynamic state changes during the progression of PAH. Differences in endothelial characteristics are observed between the early and advanced stages, suggesting that the phenotype or functional state of endothelial cells varies over the course of the disease. To confirm, lung vascular tissue from IPAH and non-IPAH patients was analyzed by IF staining, showing strong HO-1 fluorescence in non-PAH endothelial layers (Figure 5G), and diminished HO-1 signals in IPAH samples. Animal experiments (Figure 5H) confirmed this trend. IF staining of PAECs from normal and PAH rats (Figure 5I) showed reduced HO-1 in PAH, supporting the bioinformatics findings. These results suggest that PAECs are critical in PAH initiation and progression, with reduced HO-1 expression linked to endothelial dysfunction and disease progression.

### MSCs-HO-1 significantly inhibits the inflammatory response of PAECs and improves endothelial dysfunction in PAH rats

**Figure 6:**
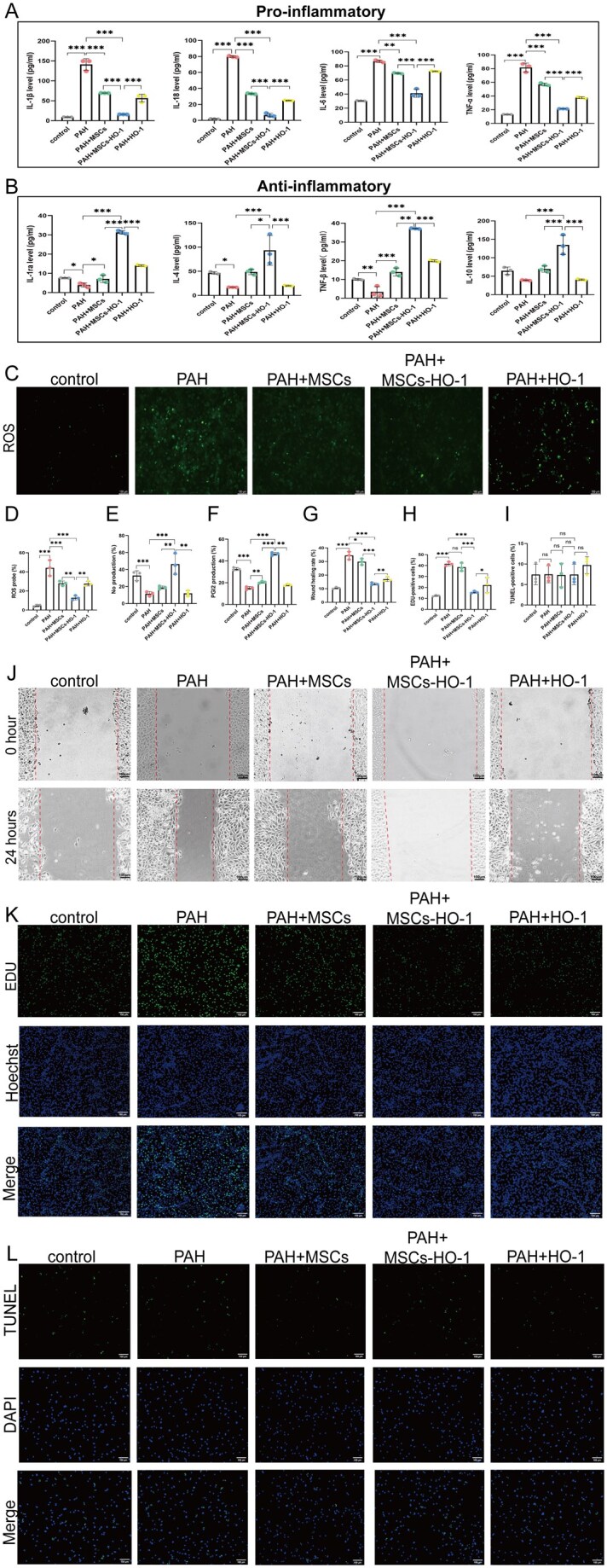
MSCs-HO-1 ameliorates inflammation, oxidative stress, and cellular dysfunction in PAECs induced by PAH. (A, B) ELISA was used to measure the levels of pro-inflammatory cytokines (IL-1β, IL-6, IL-18, and TNF-α) and anti-inflammatory cytokines (IL-10, TGF-β, IL-4, and IL-1Ra) in PAECs from different experimental groups. (C, D) ROS levels in PAECs were detected using the DCFH-DA probe (C); bar graph shows the relative ROS content in each group of cells (D), scale bar = 100 μm. (E, F) ELISA kits were used to measure the levels of NO (E) and PGI_2_ (F) in the cell culture supernatants of PAECs. (G, J) Wound-healing assay was performed to assess the migratory capacity of PAECs, with images taken at 0 and 24 h to observe the healing of the scratch area, scale bar = 100 μm. (H, K) EdU incorporation assay was used to evaluate PAEC proliferation activity, scale bar = 100 μm. (I, L) TUNEL staining was used to detect apoptosis levels in PAECs from different groups, scale bar = 100 μm. Cell experiments: *n* = 3 per group, with each experiment independently repeated three times. Differences were considered statistically significant at *P *< .05. *P < .05, **P < .01, ***P < .0001, ns *P *> .05.

To validate the mechanism of MSCs-HO-1, functional assays were conducted in rat PAECs. ELISA (Figure 6A and B) showed elevated pro-inflammatory cytokines (IL-1β, IL-6, IL-18, TNF-α) and reduced anti-inflammatory cytokines (IL-10, TGF-β, IL-4, IL-1Ra) in PAECs from the PAH group, indicating an activated inflammatory state. MSC treatment reduced pro-inflammatory cytokines and partially restored anti-inflammatory cytokines, with MSCs-HO-1 showing the most pronounced effects, returning inflammation levels closest to normal controls. While HO-1 overexpression and MSCs alone suppressed inflammation, their effects were less significant than the MSCs-HO-1 combination, highlighting MSCs-HO-1 as the most effective anti-inflammatory strategy. PAEC dysfunction, linked to oxidative stress, disrupts redox balance and inhibits NO and PGI_2_ synthesis. This ROS-mediated reduction in NO and PGI_2_ is a key feature of endothelial dysfunction in PAH.^27^^,^^28^ To assess the role of therapeutic interventions in oxidative stress-related endothelial dysfunction, ROS, NO, and PGI_2_ levels in PAECs were measured. ROS fluorescence (Figure 6C and D) was significantly reduced in the MSCs-HO-1 group compared to the other treatments. NO and PGI_2_ levels (Figure 6E and F) were lower in PAH PAECs, indicating impaired vasodilation, but increased after MSC treatment, with MSCs-HO-1 showing the most pronounced recovery, surpassing normal levels. These results suggest MSCs-HO-1 enhances antioxidant defense, promoting NO and PGI_2_ production to counteract oxidative stress. Wound-healing (Figure 6G and J) and EDU (Figure 6H and K) assays showed MSCs-HO-1 suppressed abnormal PAEC migration and proliferation, correcting pathological activation. TUNEL assay (Figure 6I and L) showed no significant apoptosis, indicating an anti-apoptotic state in endothelial cells. In conclusion, MSCs-HO-1 effectively suppressed inflammation and restored endothelial cell function in PAH.

### RNA-seq analysis indicates that differentially expressed genes in PAECs from PAH rats treated with MSCs-HO-1 are enriched in MAPK and cytokine signaling pathways

To investigate how MSCs-HO-1 improves endothelial dysfunction in PAH, RNA sequencing (RNA-seq) was performed on PAECs from MCT-induced PAH rats (*n* = 5) and MSCs-HO-1 treated rats (*n* = 5). Supplementary Figure S2A shows the workflow for PAEC isolation and RNA extraction. Supplementary Figure S2B presents a heatmap of DEGs, indicating significant transcriptional differences between MSCs-HO-1 and PAH groups, suggesting MSCs-HO-1 remodels PAEC gene expression. GO enrichment analysis (Supplementary Figure S2C) revealed key processes related to inflammation, cell migration, and stress regulation, including the Wnt signaling pathway and ion channel activity. Supplementary Figure S2D shows a circular plot of GO enrichment, highlighting the complexity of endothelial dysfunction in PAH. KEGG analysis (Supplementary Figure S2E) identified significant enrichment in the MAPK and cytokine-receptor interaction pathways, which are linked to inflammation, cell proliferation, and endothelial dysfunction in PAH. Additionally, according to previous studies,^29^^,^^30^ these two pathways are closely associated with the mechanisms of inflammation, cell proliferation, and endothelial dysfunction in PAH. Supplementary Figure S2F shows the PPI network analysis, revealing interactions between DEGs that form a highly connected regulatory network with key nodes at its center. The results suggest that MSCs-HO-1 intervention creates a complex, coordinated signaling network with multi-pathway synergy, indicating MSCs-HO-1 improves endothelial function by integrating various signaling mechanisms, providing a foundation for future studies.

### MSCs-HO-1 alleviates the inflammatory response of PAECs under PAH conditions by inhibiting the TLR4-MAPK signaling pathway

**Figure 7:**
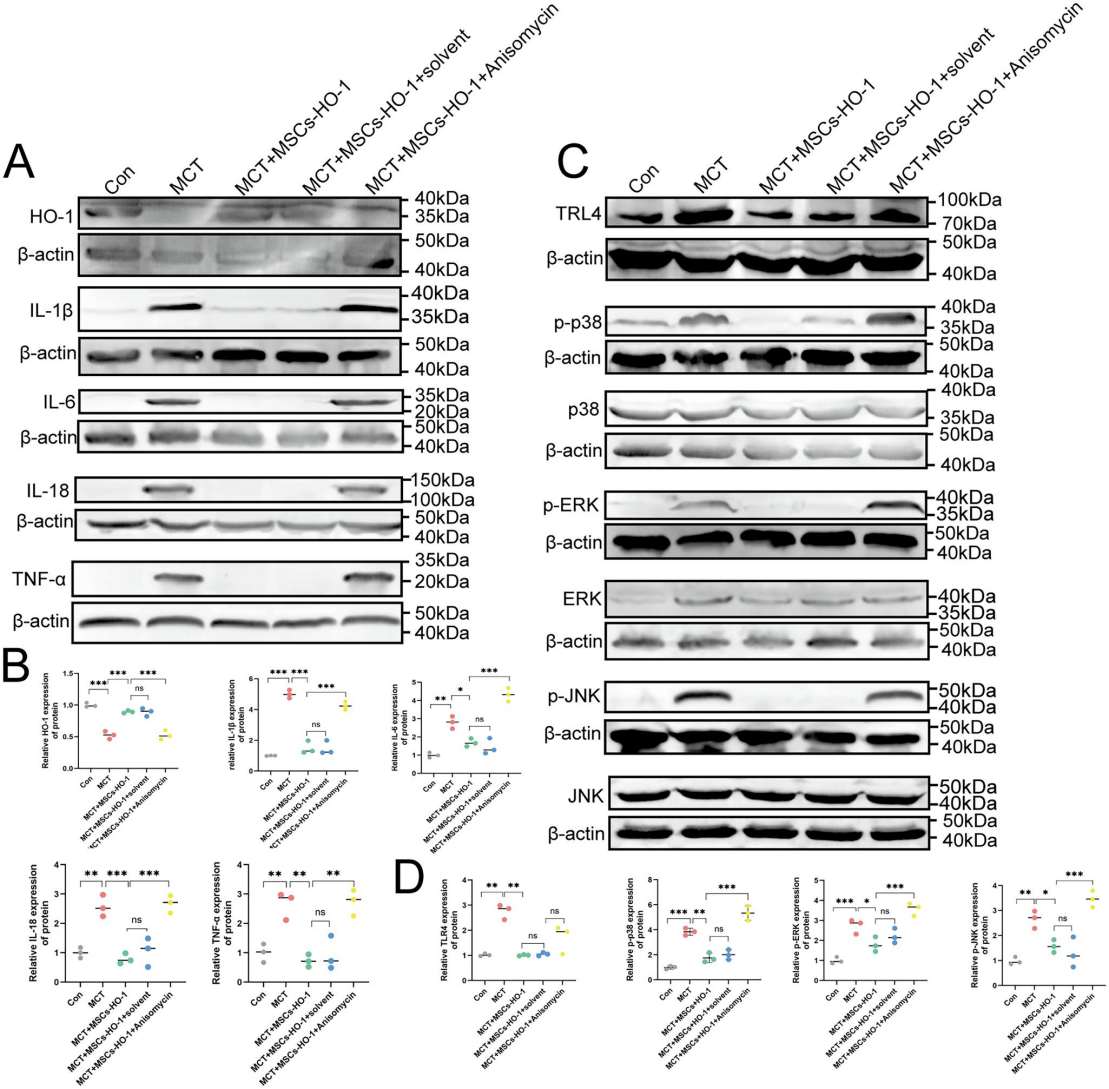
MSCs-HO-1 protects PAECs from inflammatory damage by regulating the TLR4-MAPK signaling pathway. (A, B) Representative Western blot bands (A) and band density analysis visualization (B), showing the expression of inflammation-related cytokines (IL-1β, IL-6, IL-18, and TNF-α). All target proteins and the internal control β-actin are derived from the same membrane. (C, D) Representative Western blot bands (C) and band density analysis visualization (D), showing the expression of TLR4-MAPK signaling pathway-related proteins (TLR4, p-ERK/ERK, p-p38/p38, and p-JNK/JNK). All target proteins and the internal control β-actin are derived from the same membrane. Cell experiments: *n* = 3 per group, with each experiment independently repeated three times. Differences were considered statistically significant at *P *< .05. *P < .05, **P < .01, ***P < .0001, ns *P *> .05.

Western blotting showed that MCT treatment increased inflammation-related cytokines (IL-1β, IL-6, IL-18, TNF-α), indicating inflammatory activation in PAH. MSCs-HO-1 treatment significantly reduced these cytokines, demonstrating its anti-inflammatory effect (Figure 7A and B). However, the MAPK pathway agonist Anisomycin reversed MSCs-HO-1’s effects, increasing cytokine levels to those seen in the MCT group. MCT treatment enhanced the phosphorylation of ERK, p38, and JNK, while MSCs-HO-1 inhibited this phosphorylation, blocking MAPK activation and exerting anti-inflammatory effects. Anisomycin restored these phosphorylation levels, confirming MSCs-HO-1’s effects depend on the MAPK pathway. TLR4 showed significant changes only in the MCT group, with MSCs-HO-1 still intervening in its signaling, unaffected by Anisomycin (Figure 7C and D). In summary, MSCs-HO-1 alleviates inflammation by regulating the TLR4-MAPK signaling axis and inhibiting MAPK pathway activation.

## Discussion

PAH is a progressive disease characterized by pulmonary vascular remodeling, endothelial dysfunction, and chronic inflammation, leading to increased pulmonary vascular resistance and right heart failure. Current therapies improve hemodynamics but rarely reverse vascular remodeling or inflammation.^31^^,^^32^ This study demonstrated that HO-1 is significantly downregulated in PAH patients and experimental models, contributing to increased oxidative stress and endothelial injury. As an antioxidant enzyme, HO-1 degrades heme into biliverdin and CO, exerting anti-inflammatory and cytoprotective effects. We constructed MSCs-HO-1, which showed enhanced antioxidant and anti-inflammatory capacities compared with MSCs or HO-1 alone, more effectively suppressing pro-inflammatory cytokines and increasing anti-inflammatory mediators. In both MCT and Su/Hx models, MSCs-HO-1 reduced RVSP, attenuated vascular remodeling and right ventricular hypertrophy, and improved survival. Single-cell analysis revealed that HO-1 is predominantly expressed in PAECs and markedly decreased in PAH. MSCs-HO-1 restored PAEC homeostasis by reducing ROS, improving NO/PGI_2_ signaling, and inhibiting MAPK activation. Overall, MSCs-HO-1 integrates the reparative properties of MSCs with the potent antioxidant and anti-inflammatory effects of HO-1, representing a promising cell-based therapeutic strategy for PAH.

In this study, multiple animal models were used to evaluate stem cell therapy. Compared with the chronic hypoxia model, which mainly reflects hypoxia-driven vasoconstriction and HIF-1α activation, the MCT model induces toxic endothelial injury accompanied by oxidative stress and inflammation. Because MCT metabolites directly damage pulmonary endothelial cells and promote endothelial dysfunction,^33^ this model better aligns with our focus on HO-1–mediated antioxidant regulation.

Previous studies have shown that MSCs exert protective effects in various tissue injury and inflammatory models, including myocardial infarction, liver fibrosis, acute lung injury, and renal ischemia–reperfusion, primarily through paracrine secretion, immune modulation, antioxidative activity, and tissue-repair promotion.^34–36^ However, due to the complexity of disease-specific microenvironments, MSC-based therapies often achieve suboptimal efficacy. Under ischemic, hypoxic, oxidative, or inflammatory conditions, MSCs exhibit reduced survival, impaired homing, and compromised paracrine function. Inflammatory mediators, excessive ROS, immune clearance, poor perfusion, acidosis, and extracellular matrix remodeling further limit their engraftment and sustained effects.^37^^,^^38^ Consequently, strategies to modify MSCs using physical, chemical, or genetic approaches have been developed to enhance their survival, homing capacity, immune regulation, and reparative functions in hostile microenvironments.^37^^,^^39^^,^^40^ Nevertheless, given the heterogeneity of pathological features across different diseases, a universal modification strategy is unlikely to be effective. Targeted MSC engineering should, therefore, be tailored to the specific pathological context, tissue characteristics, and therapeutic requirements of each disease.

Oxidative stress plays a pivotal role in the pathogenesis and progression of PAH. Excessive ROS reduces endothelial NO bioavailability, promotes eNOS uncoupling, and disrupts the NO–sGC–cGMP–PKG pathway, thereby aggravating pulmonary vasoconstriction and vascular remodeling. PGI_2_ acts synergistically with NO to enhance vasodilation and inhibit platelet aggregation. Accordingly, therapies that augment NO signaling or mimic PGI_2_ effects are central to current PAH treatment strategies. Thus, alleviating oxidative stress and restoring the NO–PGI_2_ signaling balance represent key therapeutic approaches in PAH.^41^^,^^42^

In line with this mechanistic framework, our study demonstrates that HO-1-modified MSCs markedly enhance antioxidant and anti-inflammatory effects in the oxidative stress microenvironment of PAH. RNA-seq analysis revealed significant enrichment of multiple pathways, including MAPK, mTOR, Wnt, calcium, cAMP, and Hippo signaling. Importantly, experimental validation identified MAPK modulation as a central mechanism underlying the protective effects of MSCs-HO-1, while the roles of other pathways remain to be clarified. Extensive evidence from oxidative stress–related disease models further supports a pivotal role of HO-1 in regulating MAPK signaling.^43^^,^^44^ In ischemia–reperfusion injury and ferroptosis, the Nrf2/HO-1 axis functions as a key antioxidant hub, with MAPK activity regulating Nrf2 nuclear translocation and HO-1 expression.^44^ Moreover, HO-1 interacts with PI3K/Akt, mTOR, and calcium signaling to influence apoptosis, inflammatory amplification, and mitochondrial homeostasis.^45^^,^^46^ Based on existing evidence, we propose that the protective effects of MSCs-HO-1 in PAH are mediated by a coordinated network centered on MAPK and integrated with oxidative stress- and inflammation-related pathways, rather than a single pathway.

This study has several limitations. First, the *in vitro* experiments were primarily performed using rat-derived PAECs and were not validated in human PAH samples, which may not fully reflect the complex pathological microenvironment and inter-individual heterogeneity of the human disease. Future studies incorporating patient-derived primary PAECs, commercially available human endothelial cells, or three-dimensional vascular organoid models would further enhance the translational relevance of our findings. Second, although RNA-seq analysis suggested the involvement of multiple signaling pathways in the protective effects of MSCs-HO-1, only the MAPK pathway was experimentally validated. The functional contributions and hierarchical interactions of the remaining pathways require further systematic investigation using gene-editing strategies and specific pharmacological inhibitors. Moreover, this study primarily focused on the functional regulation of pulmonary arterial endothelial cells, whereas the inflammatory response in PAH involves complex interactions among macrophages, neutrophils, and other immune cells in addition to endothelial cells. Future studies should establish co-culture systems of immune and endothelial cells to better elucidate intercellular communication and inflammatory microenvironmental regulation.

## Conclusion

In summary, MSCs overexpressing HO-1 show enhanced anti-inflammatory and antioxidant effects. In PAH models, MSCs-HO-1 reduce pulmonary arterial pressure, attenuate vascular remodeling, and improve right ventricular function. Mechanistically, MSCs-HO-1 regulate cytokine balance and reduce inflammation and oxidative stress in PAECs through modulation of the MAPK pathway, thereby restoring endothelial function and vascular homeostasis (Supplementary Figure S3). These findings support the therapeutic potential of MSCs-HO-1 in PAH.

## Supplementary Material

szag036_Supplementary_Data

## Data Availability

The data from this study can be obtained from the corresponding author upon request, as appropriate.
