# Supplementary material for: HO-1-modified umbilical cord MSCs alleviate pulmonary arterial hypertension by reducing inflammation and endothelial dysfunction
Source: Stem Cells Transl Med. 2026 Jun 27;15(7):szag036. doi: 10.1093/stcltm/szag036 (PMC13311674; doi:10.1093/stcltm/szag036)
Supplement: szag036_Supplementary_Data [file szag036_supplementary_data.docx]

**Supplementary Information**

**HO-1 Modification of Umbilical Cord-Derived Mesenchymal Stem Cells Alleviates Pulmonary Arterial Hypertension by Suppressing Inflammation and Dysfunction of Pulmonary Artery Endothelial Cells**

Riken Chen^¹, #^, Xing Chen^²,³, #^, Limei Liang^²,³, #^, Huan Li^¹, #^,

Liping Xu^²,³^, Dongjie Huang¹, Yong Liu^¹,⁴^,Deyi Zhou¹, Weilong Ye¹, Shuyue Zhou¹, Yihuan Su¹, Dekang Nie¹^,^⁵, Zhenzhen Zheng^¹, *^, Yan Deng^², *^

¹: The Second Affiliated Hospital of Guangdong Medical University, Zhanjiang, Guangdong, China

²: Department of Ultrasound, The First Affiliated Hospital of Guangxi Medical University, Nanning, Guangxi, China

³: Clinical Experiment Center, Guangxi Medical University, Nanning, Guangxi, China

⁴: Mudanjiang Medical University, Mudanjiang, Heilongjiang, China

⁵: Department of Neurosurgery, The Yancheng Clinical College of Xuzhou Medical University, The First People's Hospital of Yancheng, Yancheng, Jiangsu 224001, P.R. China

#: Authors contributed equally to this work.

*: Corresponding authors: Yan Deng (Email: [Dengyan@sr.gxmu.edu.cn),](mailto:Dengyan@sr.gxmu.edu.cn),) The First Affiliated Hospital of Guangxi Medical University, 6 Shuangyong Road, Nanning, Guangxi 530021, China; Zhenzhen Zheng (Email: [zhengzhenzhen2018@163.com)](mailto:zhengzhenzhen2018@163.com))

**Supplementary Table**

**TableS1. Inclusion and exclusion criteria for the IPAH and control groups**

| **IPAH group** |  |
| --- | --- |
| **Inclusion criteria:** | **Exclusion criteria:** |
| Diagnosis of IPAH confirmed by echocardiography and right heart catheterization according to current guidelines; | Secondary pulmonary hypertension caused by connective tissue disease, congenital heart disease, portal hypertension, or chronic thromboembolic disease; |
| Availability of surgically resected lung tissue and corresponding pulmonary vascular specimens; | Severe cardiac, hepatic, or renal dysfunction, malignancy, or autoimmune disorders; |
| Age between 18 and 65 years; | History of severe infection, hematologic abnormalities, or systemic inflammatory diseases; |
| Complete clinical and pathological data; | Poor tissue quality or incomplete clinical data. |
| Written informed consent obtained. |  |
| **Control group** |  |
| **Inclusion criteria:** | **Exclusion criteria:** |
| Patients undergoing resection for benign pulmonary nodules with adjacent normal lung tissue and pulmonary vascular specimens available; | Pulmonary infection, interstitial lung disease, or other pulmonary pathology; |
| No clinical, imaging, or pathological evidence of pulmonary hypertension; | History of cardiovascular, hepatic, renal, or autoimmune diseases; |
| Age between 18 and 65 years; | Presence of malignancy or severe systemic inflammatory conditions; |
| Complete clinical and pathological data; | Poor tissue preservation or incomplete clinical data. |
| Written informed consent obtained. |  |

**TableS2. Clinical characteristics of the IPAH and control groups**

| **Variables** | **Total**  **(n = 60)** | **IPAH**  **(n =30)** | **Non-PAH**  **(n = 30)** | ***P*** |
| --- | --- | --- | --- | --- |
|  |  |  |  |  |
| WHO |  |  |  | <0.001 |
| Ⅰ | 26 (43.33%) | 22 (73.33%) | 4 (13.33%) |  |
| Ⅱ | 21 (35.00%) | 7 (23.33%) | 14 (46.67%) |  |
| Ⅲ | 10 (16.67%) | 1 (3.33%) | 9 (30.00%) |  |
| Ⅳ | 3 (5.00%) | 0 (0.00%) | 3 (10.00%) |  |
| LA | 32.00 (27.00-35.30) | 27.50 (24.25-31.00) | 35.30 (34.25-35.30) | <0.001 |
| LVDd | 41.00 (32.00-44.25) | 32.00 (28.00-40.00) | 44.00 (41.25-47.75) | <0.001 |
| MVE | 55.00 (11.00-75.50) | 11.00 (10.00-52.25) | 74.80 (61.25-86.00) | <0.001 |
| MVA | 66.00 (45.75-82.25) | 47.00 (40.00-67.50) | 77.60 (66.00-87.00) | <0.001 |
| RV | 26.00 (24.00-31.00) | 30.00 (26.00-32.00) | 25.00 (21.25-25.30) | <0.001 |
| MPA | 28.00 (25.00-39.25) | 37.50 (28.25-46.25) | 27.60 (24.00-27.90) | <0.001 |
| PASP | 56.50 (35.75-84.25) | 84.00 (61.25-93.50) | 38.50 (32.00-41.20) | <0.001 |
| RA | 38.00 (20.50-41.17) | 14.00 (0.00-43.00) | 40.90 (35.00-40.90) | 0.03 |
| NT-proBNP | 424.50 (99.72-1468.25) | 829.05 (251.10-1905.75) | 272.40 (62.66-700.67) | 0.005 |

WHO, World Health Organization functional class; LA, left atrium; LVDd, left ventricular end-diastolic diameter; MVE, mitral valve E wave velocity; MVA, mitral valve A wave velocity; RV, right ventricle; MPA, main pulmonary artery; PASP, pulmonary artery systolic pressure; RA, right atrium; NT-proBNP, N-terminal pro–B-type natriuretic peptide.

**Table S3. Information on gene primers**

| **Gene** | **Direction** | **Sequence** |
| --- | --- | --- |
| *HO-1*-human | Forward primer | TCCTGGCTCAGCCTCAAATG |
|  | Reverse primer | ACGCATGGCTCAAAAACCAC |
| *GAPDH*-human | Forward primer | GTGGACCTGACCTGCCGTCTAG |
|  | Reverse primer | GAGTGGGTGTCGCTGTTGAAGTC |
| *HO-1*-mouse | Forward primer | CTAGCCCACTCCCTGTGTTTC |
|  | Reverse primer | CAGCAGTCGTGGTCAGTCAA |
| *GAPDH*-mouse | Forward primer | CCCTTAAGAGGGATGCTGCC |
|  | Reverse primer | TACGGCCAAATCCGTTCACA |
| *HO-1*-rat | Forward primer | GAACTGTGGTCGGTAGAGGC |
|  | Reverse primer | GTCAACATGGACGCCGACTA |
| *IL-1β*-rat | Forward primer | AAAATGCCTCGTGCTGTCTGA |
|  | Reverse primer | TTTGTCGTTGCTTGTCTCTCCTT |
| *IL-6*-rat | Forward primer | CACTTCAAGTCGGAGGCT |
|  | Reverse primer | AGCACACTAGGTTTTGCCGAG |
| *IL-18*-rat | Forward primer | TGCGGAGCATAAATGACCAAG |
|  | Reverse primer | TTCACAGATAGGGTCACAGCCAG |
| *TNF-α-*rat | Forward primer | GGGGATTATGGCTCAGGGTC |
|  | Reverse primer | TCTGAGACAGAGGCAACCTG |
| *IL-1 Ra*-rat | Forward primer | CGGGATGGAAATCTGCAGGG |
|  | Reverse primer | GAACCATCCTGGACAGGCAA |
| *IL-4*-rat | Forward primer | GTACCGGGAACGGTATCCAC |
|  | Reverse primer | GTGAGTTCAGACCGCTGACA |
| *IL-10*-rat | Forward primer | CATTCCATCCGGGGTGACAA |
|  | Reverse primer | GTAGATGCCGGGTGGTTCAA |
| *TNF-β*-rat | Forward primer | CAGCCTTATCTGGGTTCTCCCC |
|  | Reverse primer | GCTGGGGCTGAAGTGTAAGT |
| *GAPDH*-rat | Forward primer | GGCAAGTTCAACGGCACAG |

*HO-1*, heme oxygenase-1; *GAPDH*, glyceraldehyde-3-phosphate dehydrogenase; *IL-1β*, interleukin-1 beta; *IL-6*, interleukin-6; *IL-18*, interleukin-18; *TNF-α*, tumor necrosis factor alpha; *IL-1Ra*, interleukin-1 receptor antagonist; *IL-4*, interleukin-4; *IL-10*, interleukin-10; *TNF-β*, tumor necrosis factor beta.

**Table S4. Information on specific primary antibodies**

| **Antibody** | **Manufacturer** | **Cat No.** |  | **Dilution** |
| --- | --- | --- | --- | --- |
| HO-1 | Abcam | EPR1390Y |  | 1: 3000 |
| IL-1β | Proteintech | 16806-1-AP |  | 1: 1000 |
| IL-6 | Proteintech | 21865-1-AP |  | 1: 1000 |
| IL-18 | Proteintech | 10663-1-AP |  | 1: 1000 |
| TNF-α | Proteintech | 17590-1-AP |  | 1: 3000 |
| TLR4 | Proteintech | 19811-1-AP |  | 1: 2000 |
| P38 | CST | # 9212 |  | 1: 5000 |
| p-P38 | CST | # 4511 |  | 1: 2000 |
| ERK | CST | # 4695 |  | 1:1000 |
| p-ERK | CST | # 9211 |  | 1: 1000 |
| JNK | CST | # 9252 |  | 1: 2000 |
| p-JNK | CST | # 4668 |  | 1:1000 |
| β-actin | Proteintech | 66009-1-Ig |  | 1: 10000 |

HO-1, heme oxygenase-1; IL-1β, interleukin-1 beta; IL-6, interleukin-6; IL-18, interleukin-18; TNF-α, tumor necrosis factor alpha; TLR4, Toll-like receptor 4; p38, p38 mitogen-activated protein kinase (p38 MAPK); p-p38, phosphorylated p38 MAPK; ERK, extracellular signal–regulated kinase; p-ERK, phosphorylated ERK; JNK, c-Jun N-terminal kinase; p-JNK, phosphorylated JNK; β-actin, beta-actin.

**Supplementary Figure**


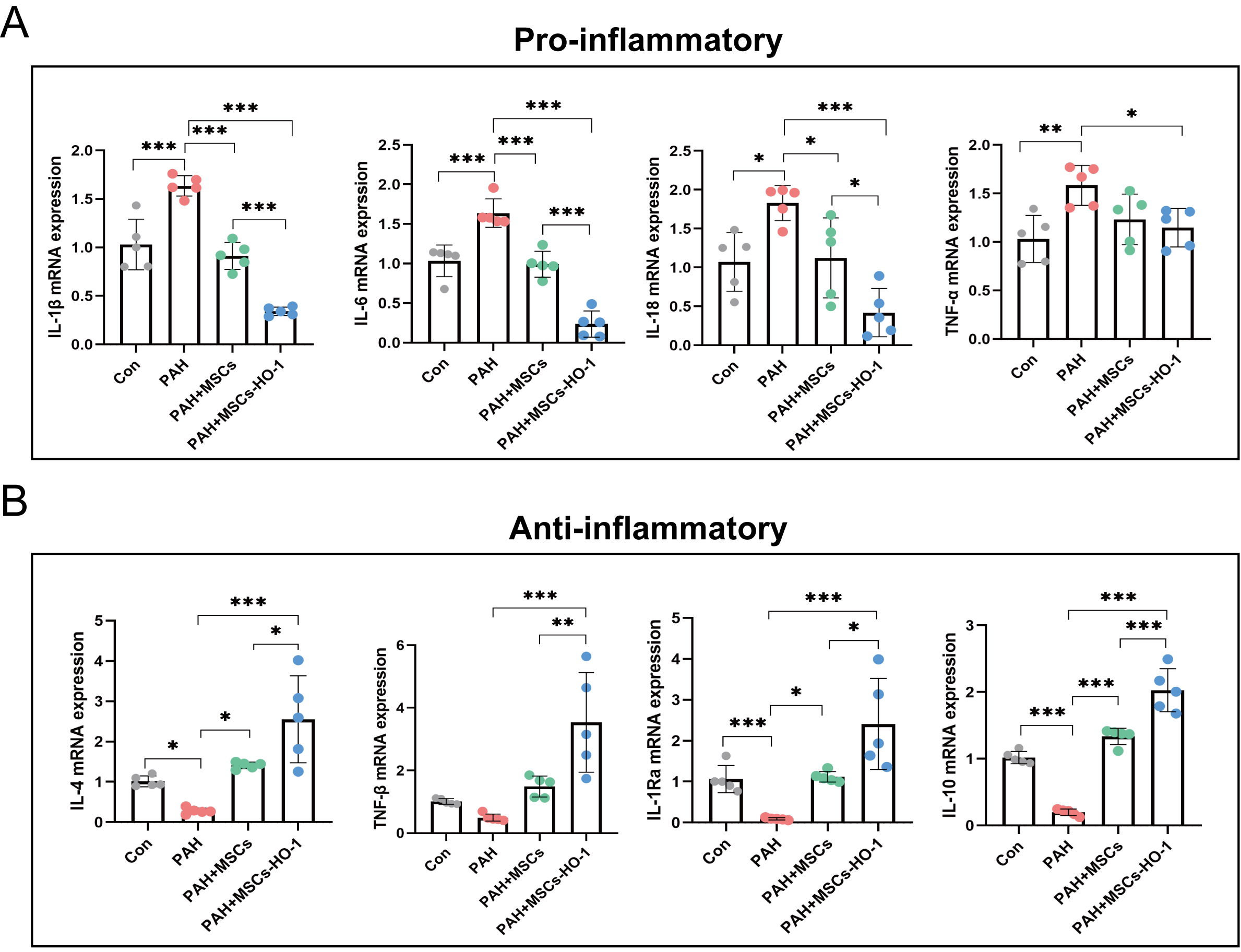


**Fig.S1. MSCs-HO-1 exhibit enhanced immunoregulatory effects, effectively improving the inflammatory environment induced by PAH.**

(A) RT-qPCR analysis of mRNA expression levels of pro-inflammatory cytokines *IL-1β*, *IL-6*, *IL-18* and *TNF-α* in the lung tissue of rats from each experimental group.

(B) RT-qPCR analysis of mRNA expression levels of anti-inflammatory cytokines *IL-4*, *TNF-β*, *IL-1Ra* and *IL-10*, and IL-1Ra in the lung tissue of rats from each experimental group.

Animal experiments: n = 5 per group, with each experiment independently repeated three times. Differences were considered statistically significant at *P* < 0.05. **P* < 0.05, ***P* < 0.01, ****P* < 0.0001, ns *P* > 0.05.


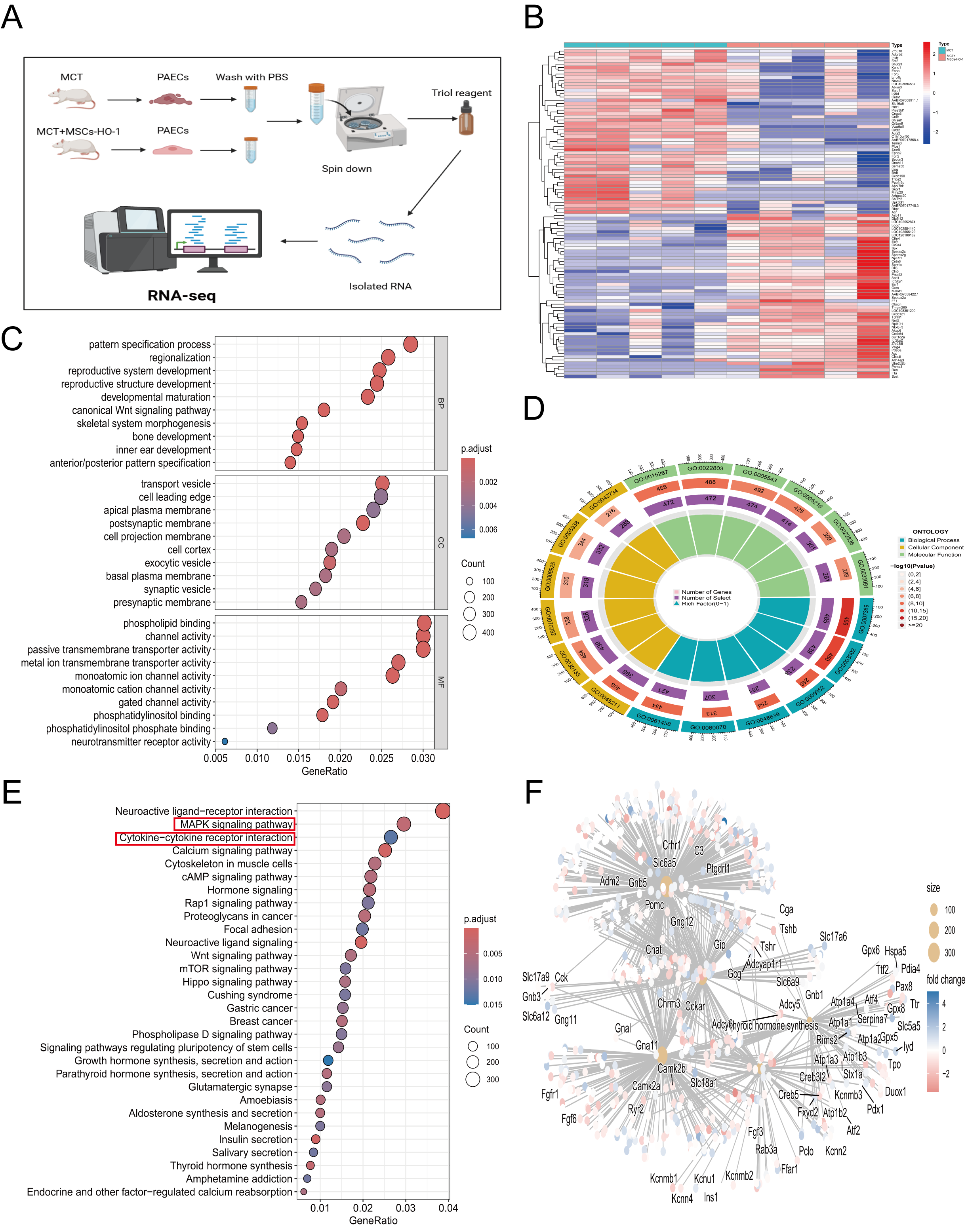


**Fig. S2. RNA-seq analysis of the effect of MSCs-HO-1 treatment on the transcriptome of PAECs in PAH rats and its potential signaling pathways.**

(A) Schematic diagram of the experimental workflow: PAECs were isolated from MCT-induced PAH rats and MSCs-HO-1 treated rats, RNA was extracted, and RNA-seq was performed (MCT group: n = 5; MCT+MSCs-HO-1: n = 5).

(B) Heatmap showing the expression profile changes of DEGs between the PAH group and MSCs-HO-1 treatment group.

(C) GO enrichment analysis to identify the major functional categories of DEGs in biological processes (BP), cellular components (CC), and molecular functions (MF).

(D) GO circular plot further illustrating the enrichment distribution of DEGs across the three major functional categories: Biological Process, Cellular Component, and Molecular Function.

(E) KEGG pathway enrichment analysis, showing significant enrichment of DEGs in key signaling pathways such as the MAPK signaling pathway and cytokine-receptor interaction pathway.

(F) PPI network interaction diagram, where node color reflects the upregulation or downregulation of gene expression, and node size represents the importance of each gene within the network.


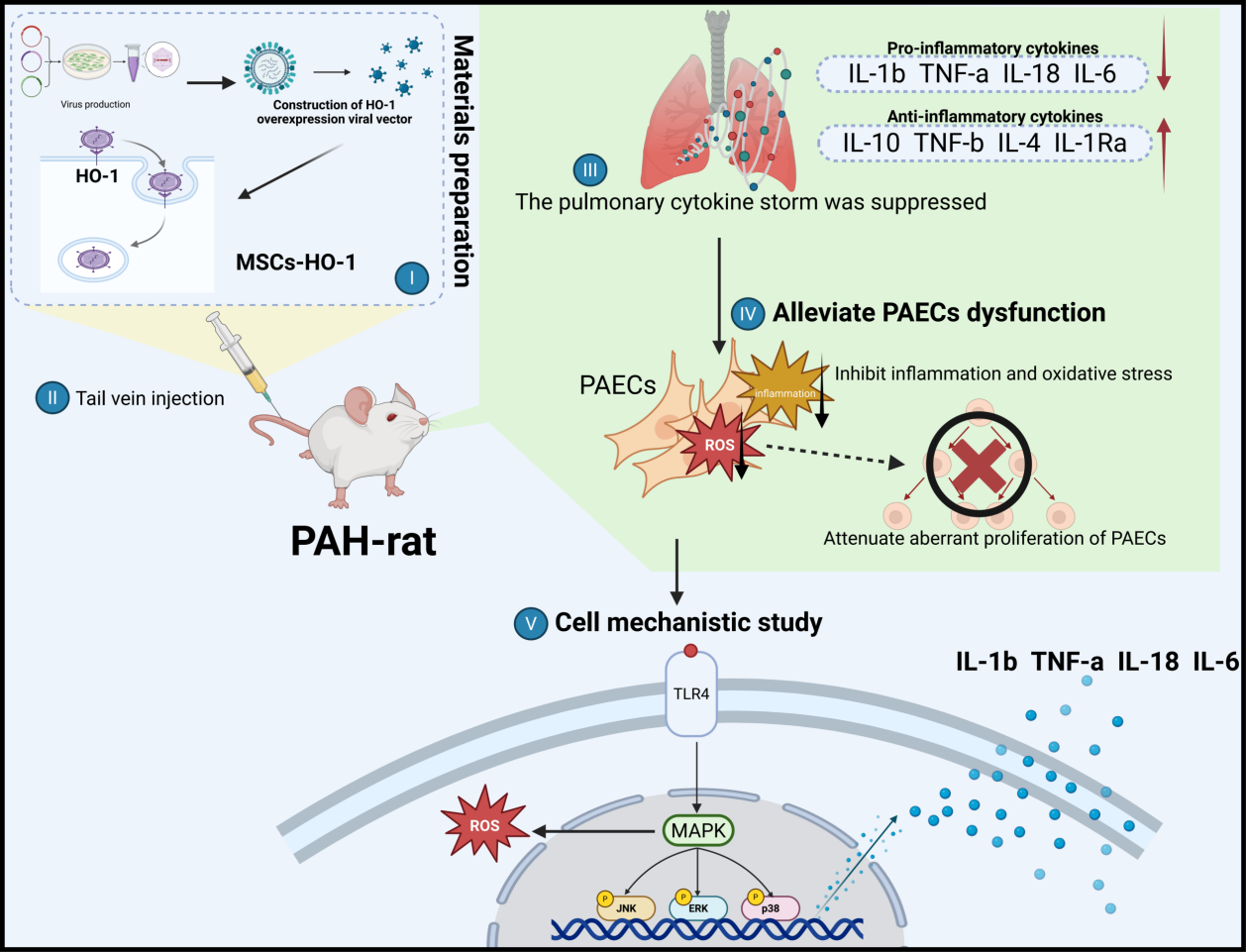


**Fig. S3. Mechanism diagram of this study**
